# Metagenome reveals caprine abomasal microbiota diversity at early and late stages of *Haemonchus contortus* infection

**DOI:** 10.1038/s41598-023-29096-9

**Published:** 2023-02-11

**Authors:** Hadeer M. Aboshady, Alice Choury, Laura Montout, Yoann Félicité, Xavier Godard, Jean-Christophe Bambou

**Affiliations:** 1 Faculty of Agriculture, Cairo University, Giza, Egypt; 2 INRAE, ASSET, 97170 Petit-Bourg, Guadeloupe France; 3 INRAE, Plateforme Tropicale d’Expérimentation sur l’Animal, 97170 Petit Bourg, Guadeloupe France

**Keywords:** Parasite host response, Metagenomics

## Abstract

*Haemonchus contortus* is one of the most detrimental gastrointestinal nematode parasites for small ruminants, especially in tropics and subtropics. Gastrointestinal nematode and microbiota share the same microhabitat; thus they interact with each other and their host. Metagenomics tools provide a promising way to examine the alterations in the gastric microbial composition induces by gastrointestinal parasites. In this study, we used metagenomics tools to characterize the impact of *H. contortus* infection on the caprine abomasal microbiota at early and late stage of infection and compared it with non-infected control. Our results showed that *H. contortus* infection caused a significant increase in abomasal pH at early (7 days post-infection) and late stage of infection (56 days post-infection). The analysis of alpha and beta diversity showed that the microbiota diversity both in number and in proportion was significantly affected at early and late stage of infection. All microbiota classes are impacted by *H. contortus* infection but *Clostridia* and *Bacteroidia* are more concerned. In infected animals, the genera Prevotella decreased at 7 and 56 days post-infection. Here we showed that the abomasal microbiota was significantly affected early after *H. contortus* infection, and these changes persist at late stage of the infection.

## Introduction

*Haemonchus contortus* is a major gastrointestinal nematode that affects small ruminant health and welfare and consequently production performances, especially in tropics and subtropics regions of the world. The pathophysiology of *H. contortus* infection is well described, with notably changes of biochemical properties of the gastrointestinal environment. Indeed, like all nematode parasites colonizing the gastrointestinal tract, *H. contortus* manipulates its microhabitat to favor its survival, increase its reproduction, and maintain its ecological niche restriction^1,2^.

The regulation of the gastric pH, is a key physiological mechanism for the maintenance of the gastrointestinal homeostasis. In ruminants, many studies showed that gastrointestinal nematodes infection are associated with increased abomasal pH and consequently increase anaerobic microbial masses in the abomasum^1,3–6^. A strong positive correlation between egg excretion and increased abomasal pH has been shown very earlier in *H. contortus* infected lambs lambs^7^.

All mammals shelter a great variety of commensal microbes that notably colonize mucosal surfaces of different tracts including the digestive, respiratory and reproductive tracts^8^. Gastrointestinal nematode and intestinal bacteria share the same microhabitat, thus they interact with each other and their host. Interestingly, in humans an increased diversity of the gut microbiota was associated with helminth colonization, suggesting a role of these parasites on the diversity, structure and function of the intestinal bacterial community^9^. Therefore, three-way interactions between the host, gut microbiota, and parasites should be closely examined in order to gain a holistic understanding of host-gastrointestinal parasites relationships.

Metagenomics tools provide a promising way to examine the alterations in the gastric microbial composition induced by gastrointestinal parasites. Recently, abomasal and rumen microbial community have been examined in sheep during early and late stages of *H. contortus* infection^2^. It has been found that *H. contortus* infection plays a decisive role in shaping composition and diversity of abomasal and ruminal microbial communities.

The changes in the caprine abomasal microbial composition induced by *H. contortus* at late infection have been recently investigated^6^. An increase in the bacterial load together with a reduced abundance of the Archaea in the abomasum was reported, nevertheless the infection did not seem to affect microbial diversity^6^. No investigation compared early (larval-stage) and late (adult-stage) *H. contortus* infection on the caprine microbial community. The present study seeks to explore the impact of early and late *H. contortus* infection on the abomasal microbial community.

## Materials and methods

All measurements and observations on animals were carried in accordance with ARRIVE guidelines and with the current law on animal experimentation and ethics, and approved by the French Ministry of Agriculture (authorization number: HC-69-2014-1) after evaluation by the Animal Care and Use Committee of French West Indies and Guyana (Comité d’Ethique en Matière d’Expérimentation Animale des Antilles et de la Guyane, C2EA-69).

### Animals, management and experimental design

This experiment was performed at the experimental farm INRAE PTEA (Plateforme Tropicale d’Expérimentation sur l’Animal) in Guadeloupe (16° 20′ North latitude, 61° 30′ West longitude). Since its foundation in 1980, the experimental goat flock owned by the INRAE PTEA is reared in this experimental farm. The experimental herd of Creole goats (breeding goats and their offspring) is routinely raised on tropical pastures year-round and thus, naturally infected mainly with two gastrointestinal nematode, *H. contortus* and to a lesser extent *Trichostrongylus colubriformis*. The experimental design was in part similar to that previously described by our team^10^. To avoid the variability of natural gastrointestinal nematode infection at pasture, a total of 22 unrelated male kids from 8 distinct sire families, were reared indoor and fed with parasite-free hay. At 4- and 6-months-old (10.62 ± 1.9 kg and 14.21 ± 2.4 kg live weight respectively), all the kids were experimentally infected with a single dose of 10,000 *H. contortus* L3 with a 10 ml syringe containing 10 ml of a suspension of L3 at 1000 L3/ml in tap water. Each experimental infection lasted 5 weeks, and was terminated with the drenching of the kids with moxidectine (Cydectine®, Fort Dodge Veterinaria S.A., Tours, France, 300 µg/kg). The faecal egg counts (FEC) were measured at week 4 and 5 post-infection. Approximately 10 g of faeces were collected in plastic tubes directly from the rectum of each animal, and transported from the experimental facility to the laboratory in refrigerated vials. The samples were individually analyzed using a modified McMaster method for determination of FEC, expressed as the number of eggs/g faeces. The mean FEC for the first and the second challenge, at 4 and 6-month-old were 4267 ± 915 and 3471 ± 718, respectively. At 9 months old (18.38 ± 3.2 kg liveweight), after a rest period of 6 weeks after drenching at the end the second challenge, 16 animals were experimentally infected for the third time with a single dose of 10,000 *H. contortus* L3 as described above, and 6 were not infected (non-infected control). At 7 days post-infection (dpi) then 56 dpi, 10 animals (7 dpi and 56 dpi groups, respectively) were humanly euthanized in accordance with the procedures authorized in animal experiments. The non-infected control animals (n = 6) were euthanized the same day than the 56-dpi group. The method used was a captive bolt stunning gun against the head of the animal, followed by exsanguination. A sample of the abomasal content (200 mg) was placed in a sterilized cryotube and snap-frozen in liquid nitrogen, then stored at − 80 °C for DNA extraction. Thereafter, the pH of the abomasal contents was measured and to recover all the parasites established, the abomasum was washed with warm distilled water and scraped with a microscope slide. The contents and the wash water were stored at 4 °C until counting. The parasites were collected, counted and sorted according to their maturity and sex.

### DNA extraction and sequencing of 16S rRNA genes

Individual abomasal DNA was extracted from approximatively 200 mg of frozen abomasal contents. All the samples were processed at the same time. Samples were incubated at 70 °C for 1 h with 250 µL of guanidine thiocyanate buffer with the following composition: 4 M guanidine thiocyanate–0.1 M Tris (pH 7.5) and 40 µL of 10% *N*-lauroyl sarcosine–0.1 M phosphate buffer (pH 8.0) and 500 µL of 5% *N*-lauroyl sarcosine. One volume (750 µL) of 0.1-mm-diameter silica beads (Sigma-Aldrich, Saint-Louis, USA) was added and the tubes were shaken for 10 min. at the maximum speed of a MM200 Mixer Mill (Retsch, Haan, Germany). The tubes were then vortexed and centrifuged at 14,000 rpm for 5 min at 4 °C. The supernatants were recovered and 30 µL of Proteinase K (Chemagic STAR DNA BTS kit, Perkin Elmer, Waltham, USA) were added. Thereafter, samples were incubated for 10 min. at 70 °C in a Multi-Therm shaker at 250 rpm (Benchmark Scientific, Sayreville, USA), then, for enzyme inactivation for 5 min at 95 °C. The tubes were centrifuged at 14,000 rpm for 5 min. at 4 °C and the supernatants were transferred in a deepwell plate. The plate was loaded onto the nucleic acid workstation Chemagic STAR (Hamilton, Perkin Elmer, Waltham, USA) for DNA extraction, performed with the Chemagic STAR DNA BTS kit (Perkin Elmer, Waltham, USA) by the @BRIDGe platform (INRAE, Jouy-en-Josas, France) according to the manufacturer’s instructions. The DNA concentrations were measured by fluorometric quantification (Qubit) and DNA samples were stored at − 20 °C. Amplification of the V3-V4 hyper-variable region of the 16S rRNA coding gene was performed on the INRAE @BRIDGe platform. Amplicon libraries of the V3–V4 region of the 16S rRNA gene were performed according to the Illumina 16S rRNA metagenomic sequencing library preparation protocol. The primers used were: the primer 1F (5′-CTTTCCCTACACGACGCTCTTCCGATCTACGGRAGGCAGCAG-3′) and the primer 1F (5′-GGAGTTCAGACGTGTGCTCTTCCGATCTTACCAGGGTATCTAATCCT-3′). The Miseq reagent kit (Illumina Inc., San Diego, CA, USA) was used for the paired-end sequencing of the pooled libraries on an Illumina Miseq platform. At the end of the run FastQ files were generated (MiSeq Reporter software, Illumina, USA). All the methods described in this manuscript are reported in accordance with ARRIVE guidelines.

### Bioinformatics analysis

Paired-end Illumina sequences data were first demultiplexed by samples, then filtered according to their Sequence Counts, their Mean Quality score and their Adaptater content obtained with fastQC. After the pre-process, reads were analyzed using Quantitative Insights Into Microbial Ecology 2 (QIIME2), mixomics, DEseq2 and philoseq.

Reads were denoised (i.e. dereplicated), chimeras identified and quality filtered using DEBLUR in the QIIME2 software. Then, ouputs were merged and clustered into OTUs (Operational Taxonomic Units) using vsearch in QIIME2. Sequences with ≥ 97% identities were assigned to the same OTUs. The SILVA database was used to classifly each OTU into the sample.

In order to get alpha and beta diversities, OTUs were first rarified. Alpha diversity was calculated using the Shannon entropy, and beta diversity was calculated employing the Jaccard index of similarity. The level of significance was determined at *P* < 0.05.

Microbial profile data were displayed using the plotbar in ggplot2 and phyloseq packages in the R software. To visualize the impact in the microbia of each group of animals, phyla, class and families are displayed in plot bars with phyloseq. The large decrease of the abundance is observed in the plot bar with absolute abundances. Whereas in the plot bar with related abundances, the differences in proportions of certain families are highlighted. The search for components to describe and discriminate samples according to their diversity and abundance is carried out with the Partial Least Squares Discriminant Analysis (PLSda) in the mixomics software. In addition, a differential OTU abundance analysis based on the negative binomial distribution was performed, using DEseq2. The *P*-value was adjusted by the Benjamini–Hochberg method. The level of significance was determined at *P* adj < 0.05 and |Fold Change|≥ 2.

## Results

### Parasitological results

**Figure 1:**
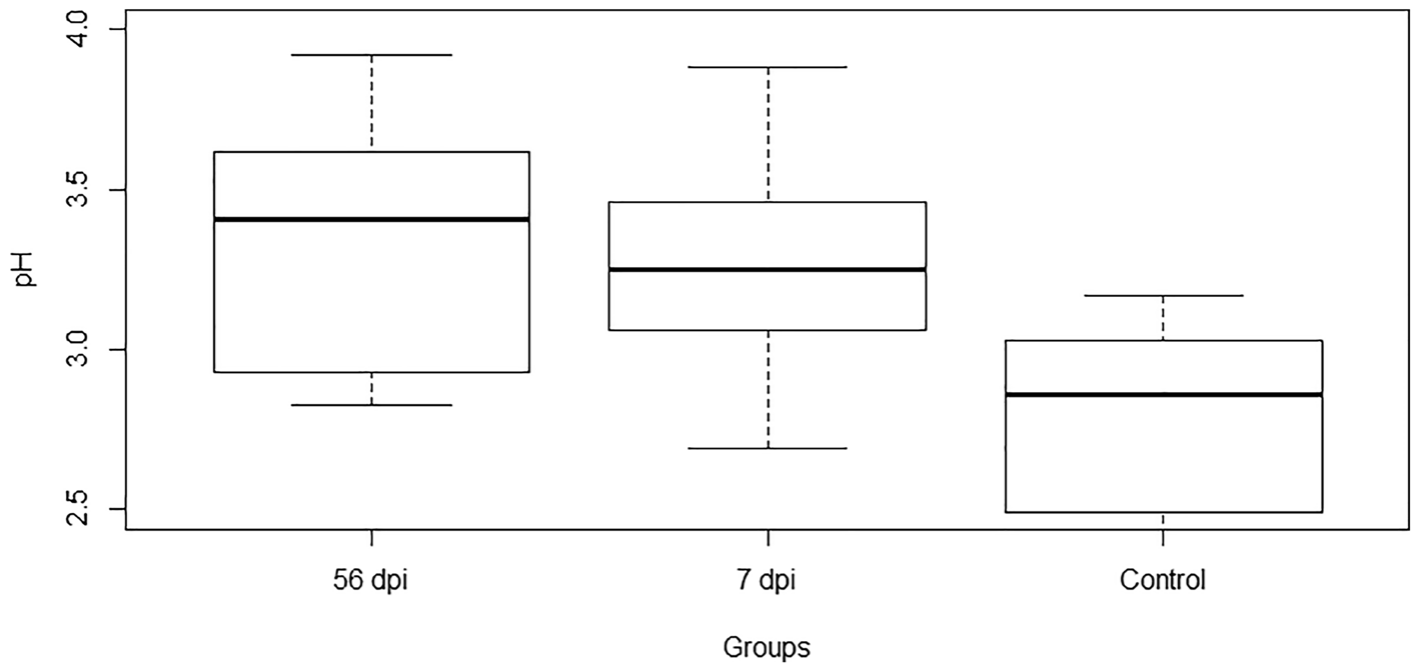
Abomasal pH of Creole goat kids comparing time points after infection with 10,000 *H. contortus* infective larvae (L3). 56 dpi (56 days post-infection), 7 dpi (7 days post-infection) and Control non-infected.

At 7 dpi, the parasite population established in the abomasum was exclusively immature (1166 ± 345 immature parasite per animal), then at 56 dpi this population was exclusively adult (112 ± 68 female, 35 ± 19 male and 142 ± 74 adult per animal, Table 1). The sex ratio was 3.2 female for 1 male. The abomasal pH was significantly increased at early (7 dpi) stage of infection compared to the uninfected control group (*P* = 0.05) and remains significantly high at late (56 dpi) stage of infection with no significant differences between early and late stage of infection (Fig. 1).

**Table 1:** Parasite burdens of Creole goat kids at 7- and 56-days post-infection with 10,000 *H. contortus* infective larvae (L3).

Parasite burden	Days post-infection		*P* value
	7	56	
Immature	1166 ± 345	0	< 0.0001
Female	0	112 ± 68	< 0.0001
Male	0	35 ± 19	< 0.0001
Total	1166 ± 345	142 ± 74	< 0.0001

### Alpha- and beta-diversity

**Figure 2:**
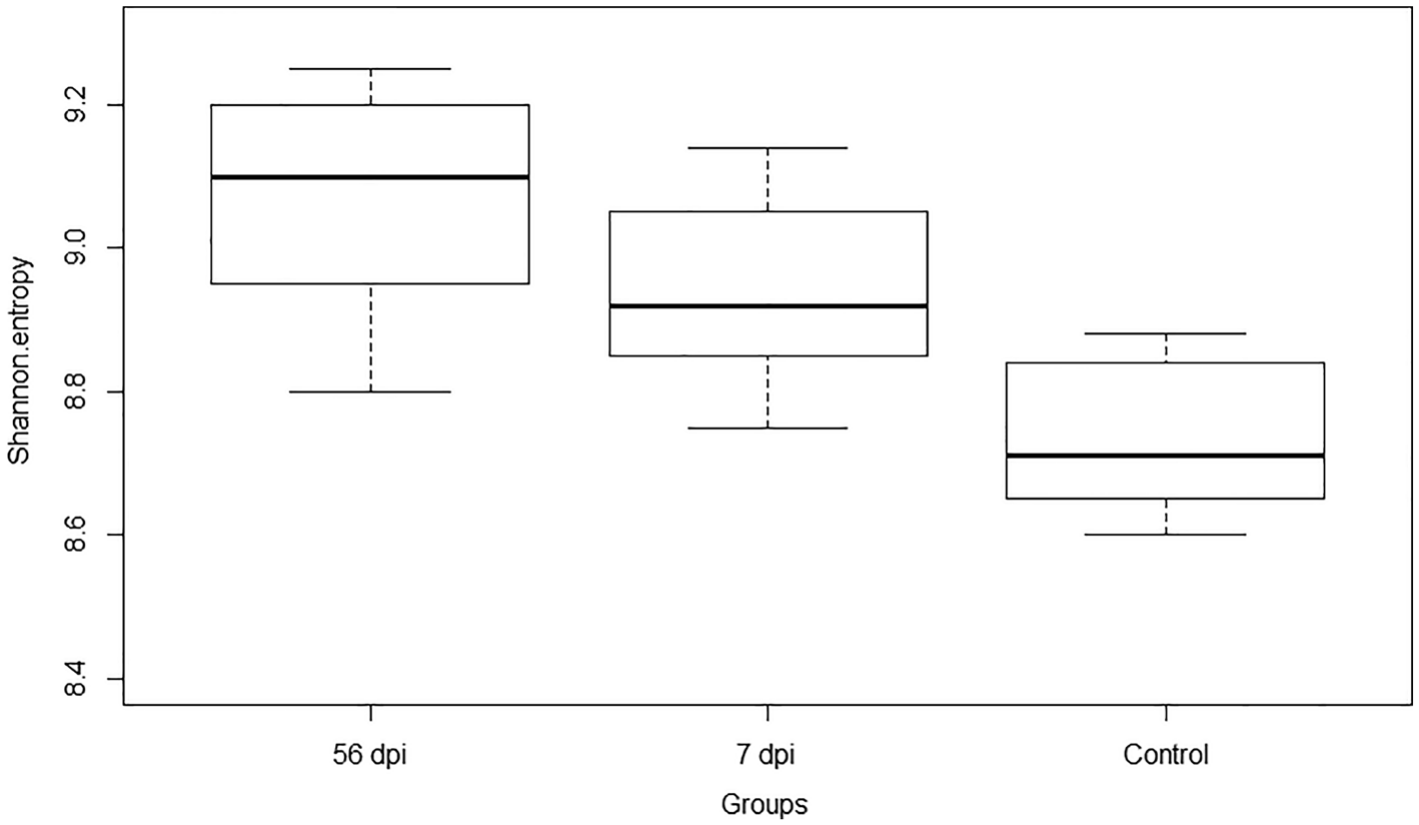
Shannon entropy for alpha diversity comparing differences between time points after infection with 10,000 *H. contortus* infective larvae (L3). 56 dpi (56 days post-infection), 7 dpi (7 days post-infection) and Control non-infected.

Shannon entropy presented an alpha diversity score more important in the non-infected control group compared with the infected groups at 7 dpi and 56 dpi (Fig. 2). The microbial community at early stage post-infection was more taxon-rich compared to that of the late stage (*P* < 0.05).

**Figure 3:**
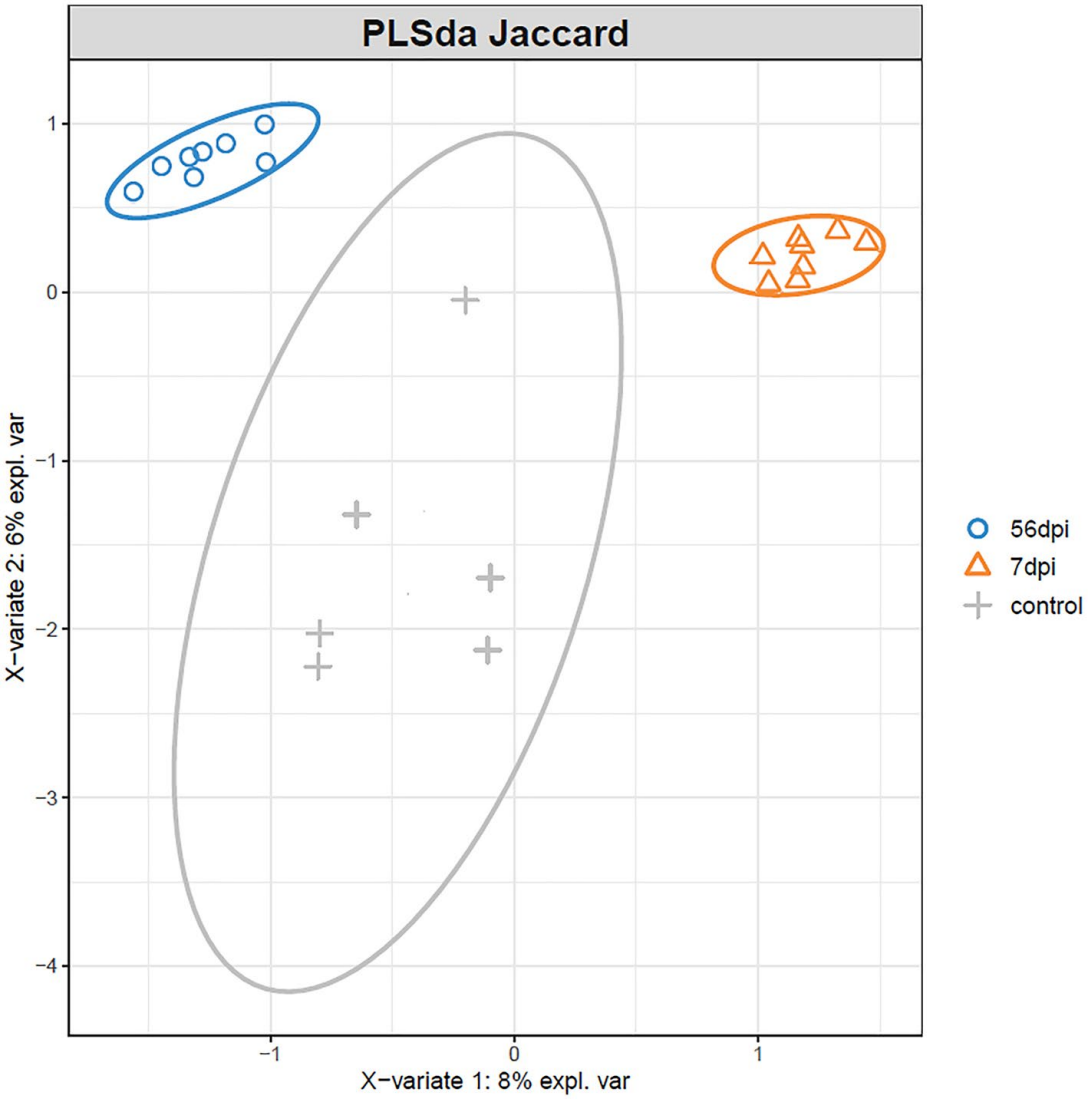
Abomasal microbiota profile of Creole goat kids comparing time points after infection with 10,000 *H. contortus* infective larvae (L3), ordinated by PLS-Discriminant Analysis (PLS-DA). 56 dpi (56 days post-infection), 7 dpi (7 days post-infection) and Control non-infected.

The Jaccard index was used to display differences between the microbial community of the non-infected control group, the early and late stage of infected goats. The matrix of distances was tested with PERMANOVA and confirms the significant differences between groups. The PLSda graph (Fig. 3) shows three separating clusters illustrating the differences in the abomasal microbiota composition between the two stages post-infection (7 and 56 dpi) compared with non-infected control goats.

### The analysis of microbial community

**Figure 4:**
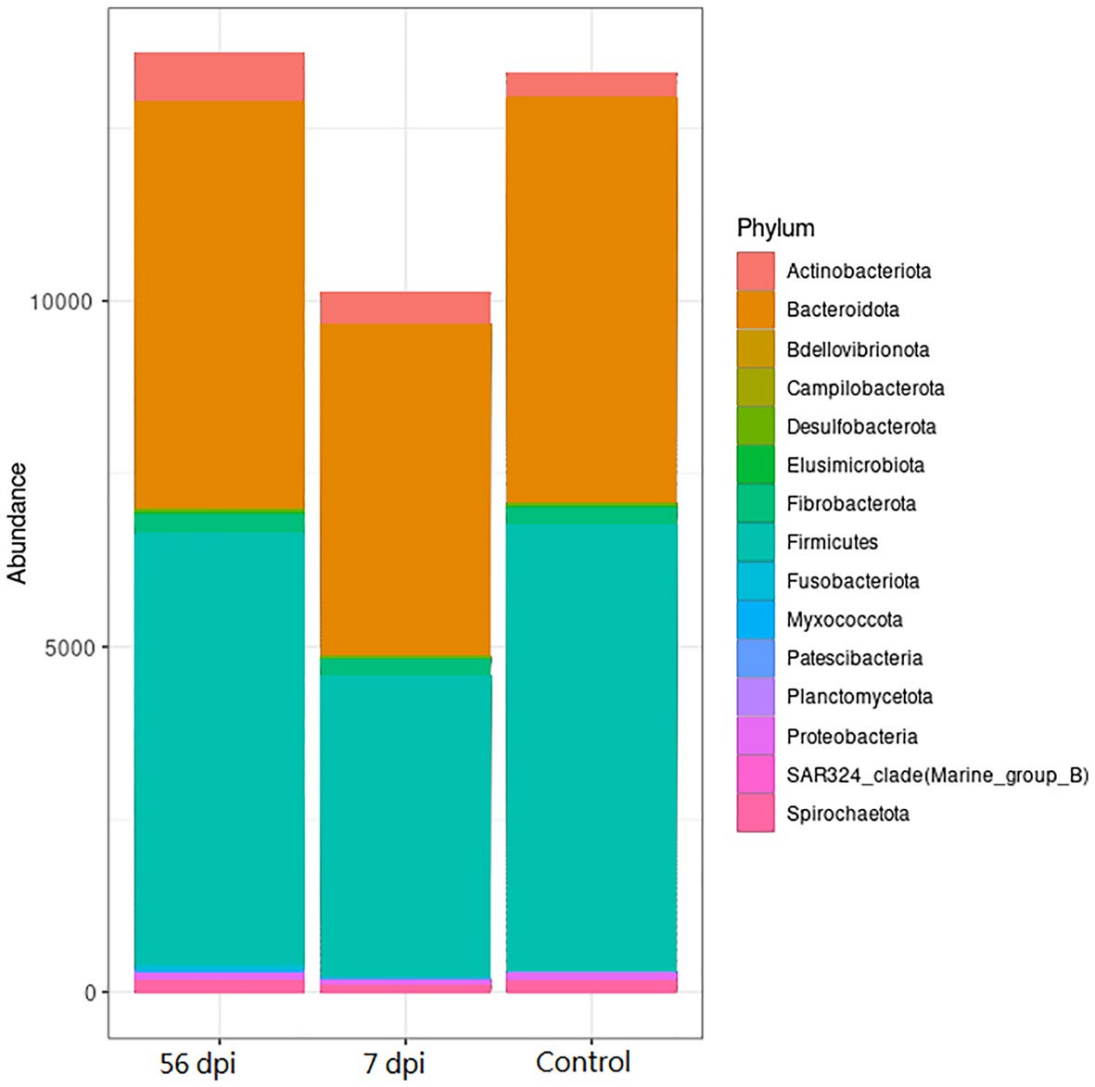
Abomasal microbial composition of Creole goat kids at phylum-level comparing time points after infection with 10,000 *H. contortus* infective larvae (L3). 56 dpi (56 days post-infection), 7 dpi (7 days post-infection) and Control non-infected.

Microbial communities are analyzed with DESeq2 to determine taxa of bacteria differentially present and abundant between each animal. The results displayed significant changes between the microbial communities of non-infected, in early (7 dpi) and late (56 dpi) infected goats, in the abundance of 15 families principally in 2 phyla: Bacteroidota and Firmicutes. The Fig. 4 shows the abundance of different phylum of the abomasal microbiota of uninfected control, early (7 dpi) and late (56 dpi) infected goats. The analysis showed a decrease, in particular of the phylum Firmicutes and Bacteroidota of the abomasal microbiota for the early-infected goats compared with the non-infected control group. Then, at 56 dpi no difference was observed between the microbiota of non-infected goats and after 56 days of infection, except for the Actinobacteriota phylum which increased.

Overall, the microbiota in all groups mainly contained the following bacterial phyla: Bacteroidota (45–49.3%), Firmicutes (40.8–45.5%), Actinobacteriota (2.4–4.6%) and Fibrobacterota (2.2–2.6%) (Fig. 4). All phyla were impacted, but Firmicutes and Bacteroidota were more concerned.

**Figure 5:**
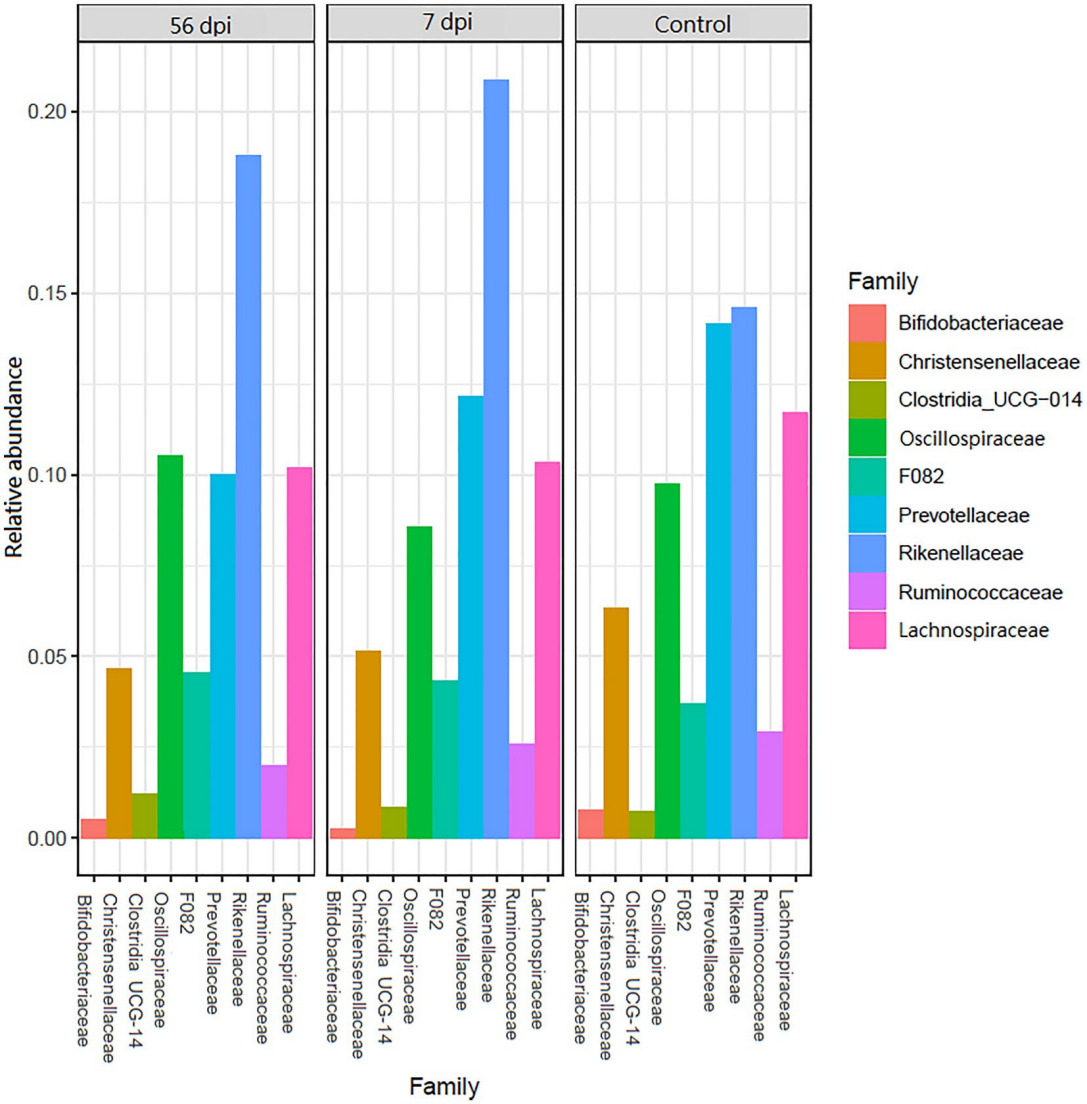
Abomasal composition of the most abundant microbial families of Creole goat kids comparing time points after infection with 10,000 *H. contortus* infective larvae (L3). 56 dpi (56 days post-infection), 7 dpi (7 days post-infection) and Control non-infected.

The Fig. 5 represents 9 selected families of the significant DESeq2 results of (*P* adj < 0.05) and shows the relative abundance in each group (56 dpi, 7 dpi and Control). The most abundant abomasal microbial families were Rikenellaceae (14.5–20.9%), Prevotellaceae (10–14.1%), Lachnospiraceae (10.4–11.7%), Oscillospiraceae (8.7–10.1%) and Christensenellaceae (4.6–6.3%) (Fig. 5). The relative abundance of Prevotellaceae decreased in the infected goats compared with non-infected control ones. Conversely, the relative abundance of Rikenellaceae increased since 7 dpi with *H. contortus*, and remained higher at 56 dpi than the value observed in the control ones (Fig. 5).

## Discussion

Colonization of the gastrointestinal tract by nematode parasites causes changes in its physiology, including an inflammatory response, an increased mucosal permeability and modifications of the biochemical properties of the luminal environment. It has been suggested that the parasites would modify their ecological niche to favor their survival and fitness. Thus, several studies reported strong evidences that gastrointestinal nematodes infections are associated with increased abomasal pH in ruminants^1–6^. The underlying mechanisms have been described as an inhibition of abomasal acid secretion mediated by gastrointestinal nematodes, associated with parietal cells dysfunction, thereby inducing elevation of the pH^11^. Similarly, in our study we showed that, in goats as early as 7 days post-infection with *H. contrortus*, the abomasal pH increased significantly and remained high at late stage of infection. These marked impacts of parasite infections on the physiology of the gastrointestinal tract are well studied in small ruminants, but the impact on the microbial communities structure and diversity, in close interaction with their local environment, remains poorly investigated.

In the present study, we showed that in goats infected with *H. contortus*, the alpha diversity of the abomasal microbiota decreased at the early stage post-infection (7 days). After 56 days post-infection, bacterial abundance increased to reach the level observed in control non-infected goats. However, microbiota diversity was lower in late-infected compared with the early-infected and the non-infected goats. These results suggested a development of some families at the expense of others, leading to a decrease in diversity. Similarly, the ovine abomasal microbiota showed significant differences in microbial diversity at 7 and 50 days post-infection with *H. contortus*^2^. The infection altered significantly the abundance of abomasal microbiota genera, nearly 98% at 7 days post-infection, and around 62% at 50 days post-infection. These results were not similar to a previous study, showing that *H. contortus* infection in goats did not affect abomasal microbial diversity at late stage post-infection (50 days)^6^. However, infection increased the abomasal bacterial load while reducing the abundance of the Archaea, which could have consequences on host protein metabolism. The impact at early stage of infection was not investigated in this study.

A decrease in Clostridia abundance was observed at early stage of infection (7 dpi) compared to the non-infected control goats, but not at 56 dpi. Interestingly, it has been shown in mice that Clostridia can induce colonic regulatory T cells, which play a central role in the suppression of inflammatory and allergic responses^12^. It is now hypothesized that, Clostridia would be involved in the maintenance of overall gut function by releasing butyrate as an end-product of fermentation through two alternative pathways: butyrate kinase and butyryl-CoA:acetate-CoA transferase^13^. Interestingly, in sheep significant differences of abomasal and ruminal Butyrivibrio abundance, a genus of the class Clostridia*,* was observed at early and late stage of *H. contortus* infection^2^. Moreover, a significant alteration of the abundance of butyrate-producing bacteria was found in goats at 50 days post-infection with *H. contortus*^6^. These different results underline the complex and dynamic relationships between gastrointestinal nematodes, the microbiota and the host mucosal immune response. Besides, it has been shown in sheep and goats that *H. contortus, Trichostrongylus colubriformis* and *Teladorsagia circumcincta* infection increased the abundance of the genera Prevotella respectively, in the ruminal, fecal or abomasal microbiota^2,6,14,15^. The increased abundance of Prevotella in the abomasum, associated with nematode infection remain unclear. Indeed, the hypothesis of a compensatory mechanism to face protein deficiency due to infection is not in line with the understanding of the mechanisms of microbiota-mediated proteo- and peptidolysis mainly performed in the rumen^16^. In the present study, our results showed a decrease of Prevotella abundance associated with *H. contortus* infection. In mice, a host-gastrointestinal parasite interaction model more investigated than ruminants, a decrease in the genera Prevotella has been observed in some studies after nematode infection^17–19^. The low number of studies conducted to date, does not allow a thorough discussion of these apparently contradictory results. Many factors could be implicated, as the different feed regimens allocated in the studies, since it has been shown recently, that particle length of neutral detergent fiber (NDF) in a feed, influenced rumen bacterial community structures^20^. The genetic background of the hosts and the different time of infection could also be implicated. It is therefore necessary to strengthen the research efforts on the close interactions between gastrointestinal parasites and the microbiota. Indeed, altogether these results suggest that gastrointestinal parasites would exert their immuno-modulatory activity partly in an indirect way by altering the structure of the microbiota.

## Data Availability

The datasets presented in this study can be found on an online public repository: https://www.ncbi.nlm.nih.gov/bioproject/PRJNA910155.
